# Deployment Readiness of Anammox for Wastewater Treatment with Potential Carbon-Saving Benefits: Environmental Risks, Monitoring Requirements and Implementation Pathways

**DOI:** 10.3390/microorganisms14092020

**Published:** 2026-09-11

**Authors:** Ya Zhou, Yi-Fei Liu, Ye Yu, Kai Wan, Yun Fang, Guo-Wei Wang, Jun-Xia Yu, Ru-An Chi, Chun-Qiao Xiao

**Affiliations:** Key Laboratory of Novel Biomass-Based Environmental and Energy Materials in Petroleum and Chemical Industry, Key Laboratory for Green Chemical Process of Ministry of Education, Hubei Key Laboratory of Microbial Transformation and Regulation of Biogenic Elements in the Middle Reaches of the Yangtze River, School of Environmental Ecology and Biological Engineering, Wuhan Institute of Technology, Wuhan 430205, China

**Keywords:** anammox, potential carbon-saving benefits, wastewater treatment, environmental risk, monitoring requirements

## Abstract

Wastewater treatment systems are under increasing pressure to improve nitrogen removal while reducing carbon emissions, yet the deployment of anaerobic ammonium oxidation (anammox) remains constrained by uncertainty about technical readiness, operational robustness, nitrous oxide (N_2_O) emissions, life-cycle carbon performance, monitoring capacity, and transferability across wastewater contexts. This study uses dynamic topic modelling and trend assessment of 998 publications from 2001 to 2025 to synthesize deployment-relevant evidence for anammox-based wastewater treatment. The results indicate that the field has shifted from reactor start-up and process-parameter optimization toward microbial regulation, mainstream process integration, coupled nitrogen-removal strategies, and intelligent control. Building on these topic-evolution patterns and reported engineering evidence, this study provides an evidence-based qualitative appraisal of deployment-readiness signals and evidence gaps, distinguishing comparatively mature side-stream applications from mainstream systems that still require monitored demonstrations, transparent N_2_O accounting, life-cycle assessment, and locally validated operating data. The study argues that anammox should be evaluated as a technology with potential but conditional carbon-saving benefits: its potential carbon-saving benefits depend on operational evidence specific to each application stage, carbon-accounting credibility, and implementation capacity, rather than assuming that research activity alone justifies broad deployment.

## 1. Introduction

Intensifying human activities and the inherent limitations of natural self-purification have led to an imbalance in reactive nitrogen, posing severe threats to global water quality and ecosystem stability. Wastewater treatment systems currently face the dual challenge of enhancing nitrogen removal efficiency while mitigating multifaceted pollutants. However, conventional heterotrophic denitrification often struggles with complex influents and is characterized by a high carbon footprint, limited resource recovery, and energy-intensive aeration, necessitating sustainable alternatives [1,2]. Driven by the global potential carbon-saving benefit transition, the anammox process has emerged as a cornerstone technology. By autotrophically converting NH_4_^+^-N and NO_2_^−^-N into N_2_ without external carbon sources and with minimal aeration [3], the discovery of anammox has revolutionized conventional nitrogen cycle theory. This paradigm shift integrates pollution abatement with carbon emission reduction.

Anammox research is currently focused on synergistic advances in systems biology and process intensification. Multi-omics methods have elucidated anammox bacteria’s metabolic pathways and regulatory mechanisms [4]. Meanwhile, anammox granular sludge technology has enabled the shift from treating high-strength side-streams to treating mainstream low-ammonia wastewater [5,6]. Despite rapid advances in the anammox process, full-scale application in mainstream systems still faces major challenges. These include incomplete understanding of microbial inhibition by complex matrices, unstable supply of crucial intermediates, and operational instability under low temperatures or fluctuating loads.

Recent studies have investigated anammox from complementary scientific and engineering perspectives. Mechanistic studies have examined microbial metabolism, functional interactions, and regulatory responses, whereas reactor and operation-oriented studies have evaluated biomass retention, NOB suppression, nitrite supply, and process control under different wastewater conditions [7,8,9]. Pilot- and full-scale studies have further assessed system performance under specific influent characteristics and operating conditions [10]. Meanwhile, literature-based analyses have characterized publication patterns, keyword relationships, research hotspots, and collaboration networks in the anammox field. Collectively, these studies have provided important evidence concerning microbial mechanisms, process configurations, operational strategies, and the overall research landscape.

However, the temporal evolution of research topics and the relationship between changes in research attention and deployment-related evidence remain insufficiently characterized. Translating this expanding body of knowledge into practical implementation requires consideration not only of process performance but also of operational robustness, greenhouse gas accounting boundaries, monitoring capacity, site-specific conditions, and technology transfer across different climatic, regulatory, and infrastructure contexts.

To address this gap, the present study constructed a structured dataset of 998 publications and applied dynamic topic modelling and trend analysis to examine the longitudinal evolution of anammox research. Specifically, this study investigated the following: (1) how research attention shifted from reactor start-up and process optimization towards microbial regulation, mainstream integration, coupled nitrogen-removal processes, and intelligent control; (2) how the prevalence and content of the identified topics changed over time; (3) how these topics relate to deployment-relevant dimensions, including operational robustness, environmental performance, monitoring requirements, and implementation conditions; and (4) which evidence gaps require further pilot and full-scale validation. Changes in topic intensity were interpreted only as changes in research attention and not as direct evidence of technological maturity or deployment readiness. Compared with studies focusing on individual mechanisms, reactor configurations, or aggregated publication and keyword patterns, the present study examines topic evolution from a longitudinal perspective. By combining dynamic topic analysis with an evidence-based qualitative appraisal, this study provides a structured analytical basis for identifying research priorities and deployment-related evidence gaps and for supporting the staged and site-specific implementation of anammox as an energy-efficient autotrophic nitrogen-removal process with potential carbon-saving benefits.

## 2. Materials and Methods

### 2.1. Dataset Construction and Eligibility Screening

**Figure 1:**
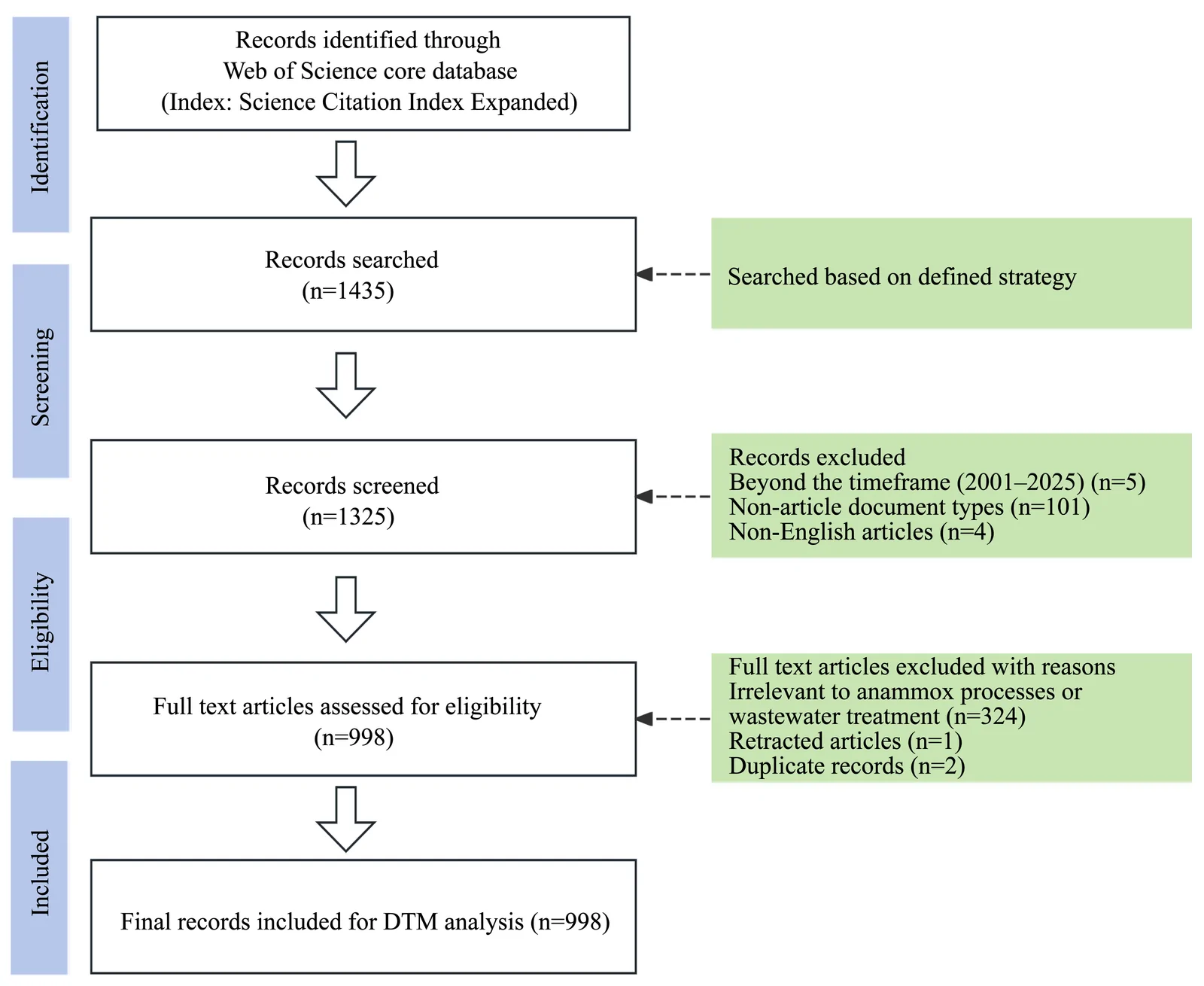
Literature screening flow chart.

The Science Citation Index Expanded (SCIE) within the Web of Science Core Collection (WoSCC) is an internationally recognized, authoritative multidisciplinary literature database. This study conducted a systematic literature search in WoSCC (SCIE). Using the advanced search function, the following search strategy was constructed: AB = (“anaerobic ammonium oxidation” OR “anammox” OR “deammonification”) AND AB = (“wastewater” OR “sewage” OR “effluent”) AND AB = (“nitrogen removal” OR “total nitrogen” OR “anammox process”). This requirement was intended to ensure that the included studies explicitly addressed anammox, wastewater treatment, and nitrogen removal, thereby improving the thematic relevance of the DTM corpus and reducing interference from studies that mentioned anammox only as background information. However, this search strategy may have excluded relevant studies that did not simultaneously state these core concepts in their abstracts. Inclusion criteria were (1) original research articles; (2) published in English; and (3) dated between 1 January 2001 and 1 December 2025. All records meeting these criteria were downloaded in plain-text format, including full record and cited references. Based on the above search results, two researchers (Y.Z. and Y.Y.) screened titles and abstracts. The exclusion criteria were as follows: (1) the study content was not directly related to the application of anammox in wastewater nitrogen removal; (2) non-research literature; (3) failure to simultaneously address the three core elements of anammox, nitrogen removal, and wastewater treatment; and (4) retracted publications. Any disagreements during the screening process were resolved through discussion with a third researcher (K.W.) to reach consensus. After duplicate removal and eligibility screening, 998 articles were retained for dynamic topic modelling and qualitative appraisal of deployment-relevant evidence (Figure 1).

### 2.2. Dynamic Topic Modelling

Dynamic topic modelling was used to identify deployment-relevant themes and their temporal changes in the structured literature dataset. Topic models extract latent thematic structures from large text corpora and are useful for comparing evidence patterns across long research periods [11]. Unlike static Latent Dirichlet Allocation, DTM incorporates time slices and can therefore capture changes in topic composition and prevalence (Figure S1). Titles, abstracts, and keywords were cleaned, tokenized, stemmed, and filtered for stop words using Gensim and NLTK in Python (version 3.11). The period from 2001 to 2025 was divided into temporal stages, and topic coherence scores were used to determine the optimal number of topics. The resulting topics were interpreted according to their dominant terms, representative records, and relevance to anammox deployment in wastewater treatment.

### 2.3. Trend Assessment

To identify recent shifts in deployment-relevant research fronts, polynomial regression analyses were performed for topic intensity from 2018 to 2025, with year as the independent variable and topic intensity as the dependent variable. Statistical significance (*p* < 0.05) was used to identify topics with increasing or decreasing research attention. These trends were interpreted as indicators of changing evidence priorities rather than direct measures of technological readiness.

### 2.4. Evidence-Based Qualitative Appraisal Framework for Deployment Readiness

To avoid treating topic prevalence as a direct proxy for deployment readiness, the DTM and trend results were interpreted through an evidence-based qualitative appraisal framework. Each topic was discussed across four deployment-relevant dimensions: reported engineering progress, operational robustness, carbon-accounting credibility, and management actionability. Reported engineering progress was appraised using topic content together with independently reported evidence of full-scale practice, monitored demonstrations, or unresolved mechanistic investigation; topic prevalence or temporal growth alone was not treated as evidence of readiness. Operational robustness was discussed by identifying reported evidence related to process stability, low-temperature operation, nitrite supply, microbial monitoring, influent fluctuation, and greenhouse gas risk, as well as substrate limitation, start-up requirements, and stability under defined monitoring conditions. Carbon-accounting credibility was considered where a topic affected energy use, chemical input, sludge production, direct N_2_O emissions, or life-cycle carbon performance, with attention to the availability, comparability, and verifiability of the reported data, the defined system boundaries, and the inclusion of relevant inputs and emissions. Management actionability was defined by whether the evidence could inform decisions by wastewater utilities, regulators, funding agencies, or technology providers, including monitoring protocols, operating standards, demonstration programmes, infrastructure planning, and site-specific deployment decisions.

This framework was used to translate the five DTM topics into deployment-relevant categories. Reactor configuration and start-up were interpreted as potential priorities for side-stream standardization and operator training; microbial metabolism and regulation were interpreted as evidence for monitoring indicators and microbial risk management; mainstream process integration was interpreted as evidence for demonstration programs and infrastructure planning; coupled nitrogen-removal technologies was interpreted as evidence for multi-pollutant treatment and nutrient-recovery management; and process parameters and intelligent control were interpreted as evidence for monitoring, reporting and verification (MRV); digital control standards; and operational data disclosure. These topic-to-category links were used to organize the available evidence and identify potential deployment priorities, rather than to establish causal relationships or assign readiness levels. The framework does not assume that increasing research intensity alone justifies broad implementation. Instead, it identifies where the literature suggests potential priorities for standardization, where independent engineering evidence remains necessary, where uncertainty warrants monitored pilots, and where further research is needed before broad deployment. No numerical readiness scores, weighting procedures, and formal validation against a technology readiness level (TRL) scale were applied. Accordingly, this framework is intended as a conceptual, evidence-based tool for qualitatively organizing deployment-related evidence and evidence gaps, rather than as a validated readiness index.

## 3. Results

### 3.1. Temporal Phase Delineation

This study divides the field’s development into four stages based on its evolutionary characteristics: foundational establishment and engineering validation; process optimization and side-stream dissemination; mainstream breakthroughs with elucidation of microbial mechanisms; and system integration and regulation. This temporal segmentation is intended to provide a historical evolutionary framework to support subsequent thematic classification and differential analysis.

### 3.2. Determination of the Optimal Number of Topics

**Figure 2:**
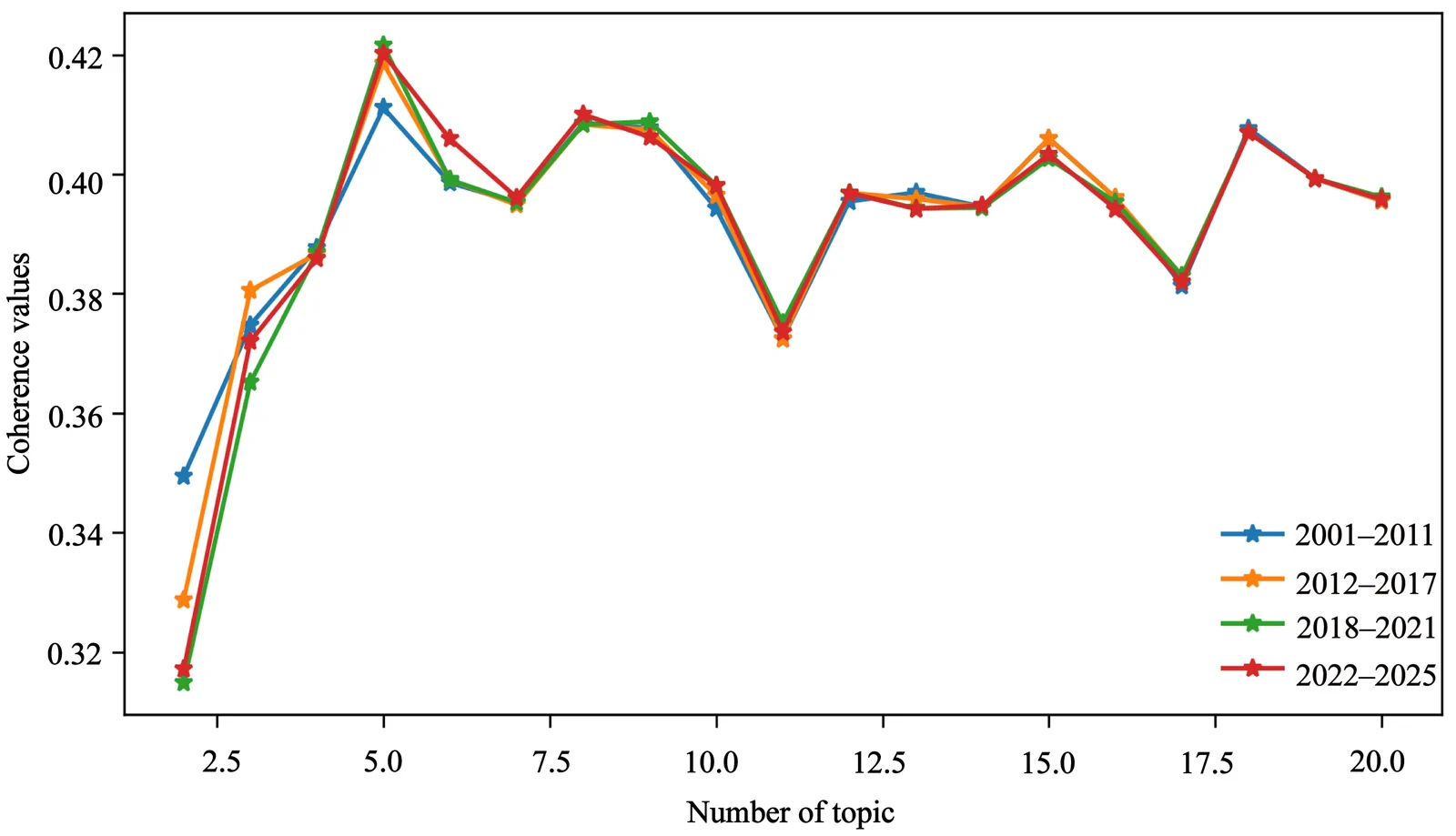
Evaluation of topic coherence scores across distinct evolutionary stages of anammox research, the temporal framework is categorized into four pivotal phases: (1) 2001–2011: Foundational establishment and engineering validation; (2) 2012–2017: Process optimization and side-stream dissemination; (3) 2018–2021: Mainstream breakthroughs with elucidation of microbial mechanisms; (4) 2022–2025: System integration and regulation.

Candidate topic numbers were evaluated within the interval K = 2–20, and topic coherence (C_V) was used as the evaluation metric. This metric quantifies a topic’s logical coherence and interpretability by measuring the semantic similarity and co-occurrence probability of the topic’s high-frequency feature words across the corpus. A higher consistency score denotes a tighter semantic focus within the topic [12]. The topic coherence results (Figure 2) show that the C_V metric rises at first and then falls as the number of topics increases, attaining a global maximum at K = 5. Therefore, this study determines the optimal number of topics to be 5 and uses this value as the fundamental parameter for subsequent analyses of topic dynamic evolution.

### 3.3. Topic Recognition

Using a DTM-based analysis of topic word distributions in the text data, the top 20 topic terms by weight were extracted for each time period (Tables S1–S4). Based on the top 10 dominant topic terms for each thematic cluster, five topic labels were identified and assigned: Topic 1 (reactor configuration and start-up); Topic 2 (microbial metabolism and regulation); Topic 3 (mainstream process integration); Topic 4 (coupled nitrogen removal technologies); Topic 5 (process parameters and control) (Table 1).

### 3.4. Topic Intensity Analysis

**Figure 3:**
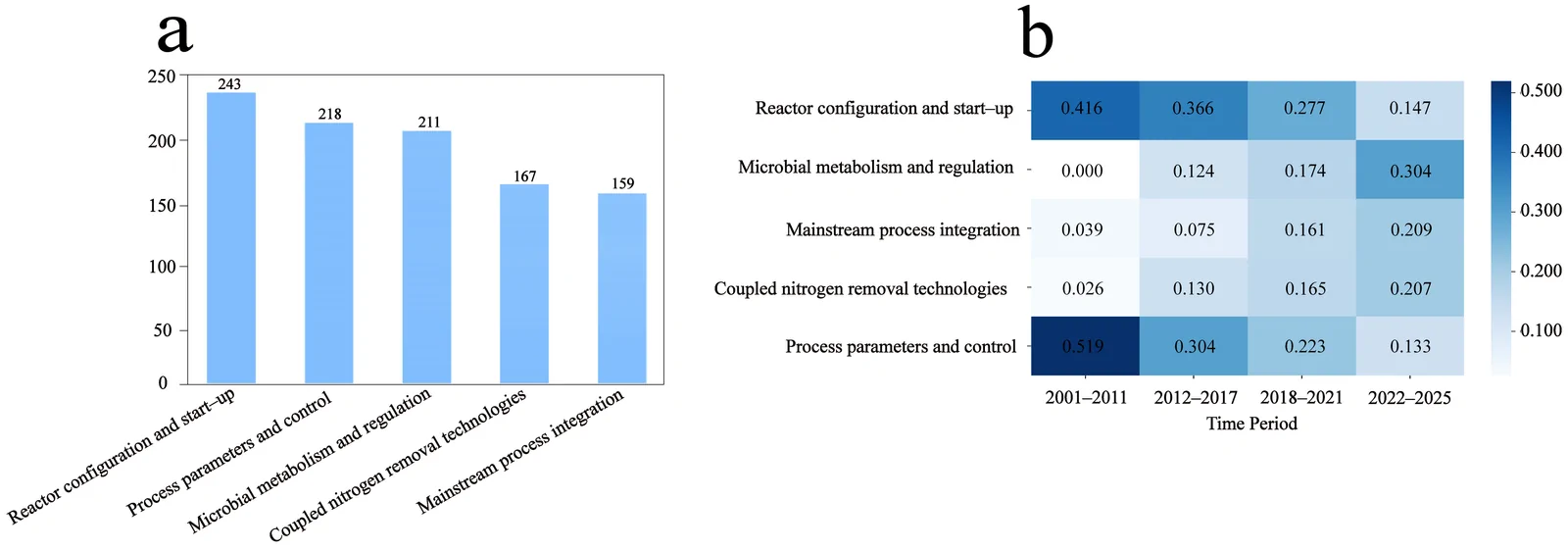
Multi-dimensional analysis of thematic intensity across the anammox research landscape. (**a**) Topic prevalence proportions: Illustrating the relative weights and quantitative distribution of individual topics within the entire corpus. (**b**) Thematic intensity heatmap: A matrix characterizing the temporal prominence and association strengths of themes across successive time slices.

Topic intensity proportions (Figure 3a) show relatively high proportions for 2001–2025 Topic 1, Topic 5, and Topic 2, while Topic 4 and Topic 3 show relatively low proportions. By calculating the topics’ intensity per stage, the research focus for each period was identified. The results (Figure 3b) indicate that the interval from 2001 to 2011 constituted a phase of foundational establishment and engineering validation for this field. Dominant topic patterns were centered primarily on Topic 1 and Topic 5, indicating that early efforts concentrated on constructing fundamental theories and initiating the preliminary transition from laboratory studies to large-scale engineering applications. From 2012 to 2017, research attention increasingly shifted towards side-stream processes. As side-stream processes grew in wastewater treatment plants, the relative topic intensities of Topics 1 and 5 decreased, and multi-pathway synergies and succession of the microbial community were focused on Topic 2, Topic 3, and Topic 4. From 2018 to 2021, the field underwent mainstream breakthroughs with elucidation of microbial mechanisms.

### 3.5. Thematic Evolution Path Analysis

**Figure 4:**
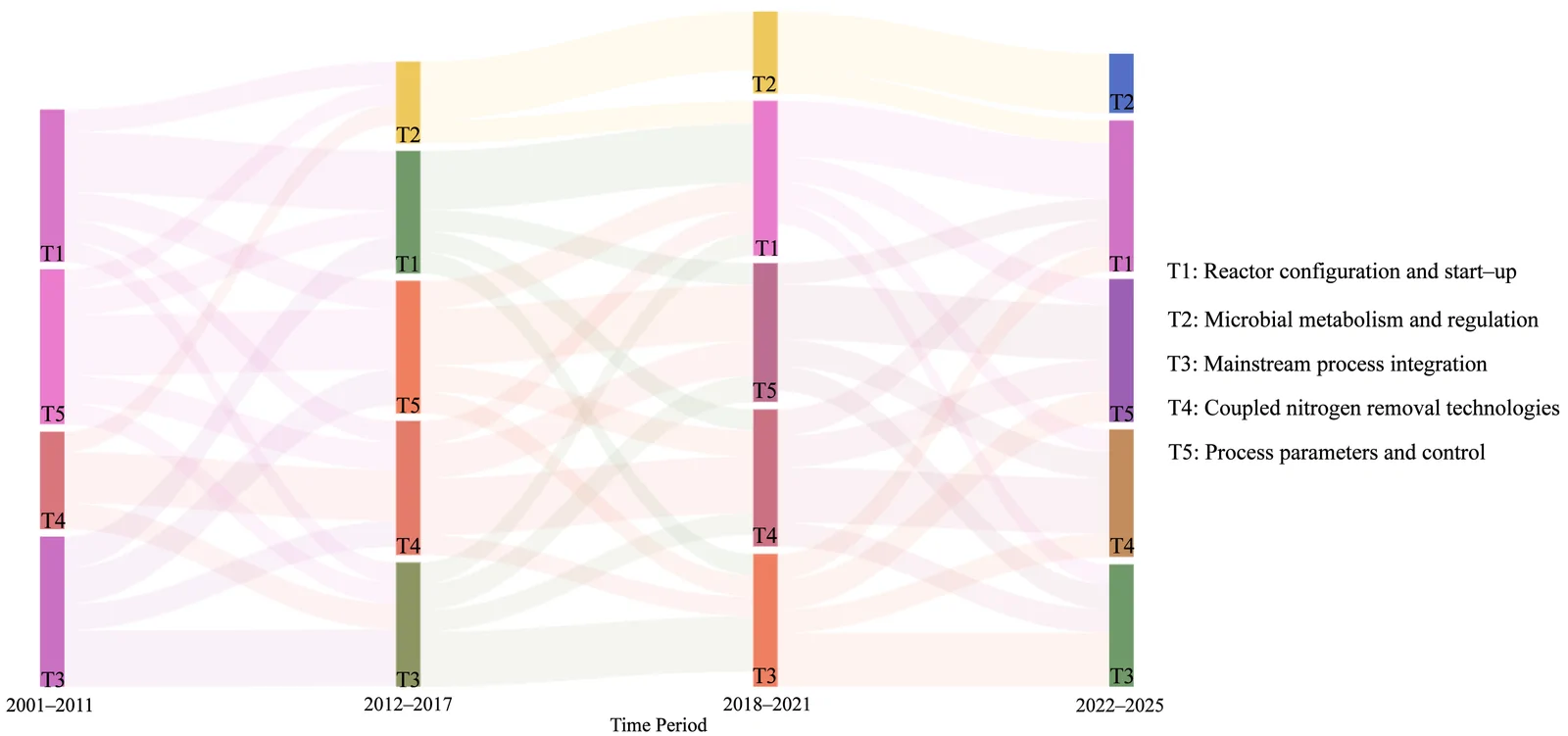
Sankey diagram of thematic evolution pathways. Nodes, links, and flows represent the identified themes, evolutionary connections, and relative thematic flow, respectively.

Thematic evolution path analysis is a quantitative method for analyzing the dynamic evolution of a research field over long periods. It identifies the successions and migrations of research dominant themes between stages and the connections that lead to thematic structural reorganization. Figure 4 shows the trends between 2001 and 2025 depending on the topic similarities between the two stages. The field evolves from macro-scale engineering construction towards micro-scale mechanism construction and towards integrated system intelligence. The initial phase was mainly concerned with Topic 1 and Topic 5, so early studies focused on engineering groundwork for anammox bacteria retention optimization of reactor configuration and operational parameters, marking the transition from laboratory discovery to engineering-scale validation. Since the activity and stability of anammox bacteria are sensitive to many operational parameters, studying these parameters is essential to achieve engineering implementation. During process optimization and side-stream dissemination stages, as side-stream high-ammonia nitrogen wastewater treatment approaches matured, the paths from Topic 1 and Topic 5 converged toward Topic 4.

### 3.6. Recent Temporal Trends in Topic Intensity

**Figure 5:**
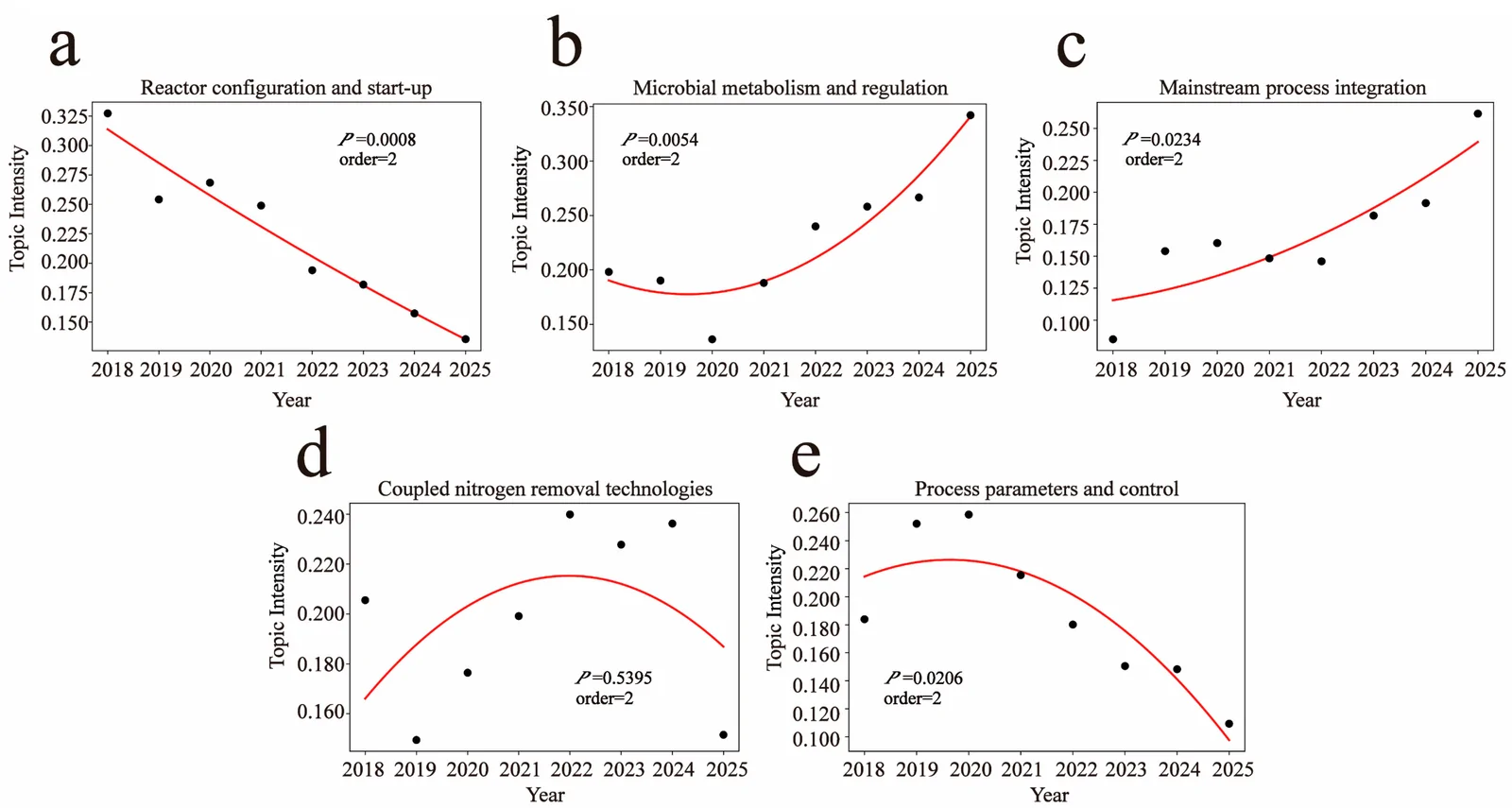
Temporal trend analysis and polynomial regression of topic intensity for each topic (2018–2025). The scattered points represent annual observations, while the solid lines denote the fitted nonlinear trajectories. (**a**) Reactor configuration and start-up; (**b**) Microbial metabolism and regulation; (**c**) Mainstream process integration; (**d**) Coupled nitrogen removal technologies; (**e**) Process parameters and control.

To explore recent changes in topic intensity from 2018 to 2025, polynomial regression models were fitted using year as the independent variable and annual mean topic intensity as the dependent variable. Trends were classified according to the direction and shape of the fitted curves and their statistical significance (*p* < 0.05). Because the analysis included only eight annual observations, it was treated as exploratory. The fitted curves and *p* values may be sensitive to individual years, model specification, temporal autocorrelation, and short-term fluctuations. Therefore, the regression results were used only to describe patterns within the observation period, rather than for causal inference, long-term forecasting, or technology-readiness assessment. Topic 2 (R^2^ = 0.8757, *p* = 0.0054; Figure 5b) and Topic 3 (R^2^ = 0.7773, *p* = 0.0234; Figure 5c) showed increasing fitted trajectories. Topic 1 (R^2^ = 0.9412, *p* = 0.0008; Figure 5a) and Topic 5 (R^2^ = 0.7883, *p* = 0.0206; Figure 5e) showed decreasing trajectories. No statistically detectable temporal trend was observed for Topic 4 (*p* > 0.05; Figure 5d). The increasing research attention to Topics 2 and 3 during 2018–2025 describes a temporal redistribution of research activity but does not, by itself, support forecasts of future trends or conclusions about technological maturity or deployment readiness.

## 4. Discussion

### 4.1. Technological Evolution and Emerging Directions of Anammox-Based Nitrogen Removal

The period from 2001 to 2011 is designated as that of foundational establishment and engineering validation. The core breakthrough in this interval was the proposal of the Completely Autotrophic Nitrogen removal Over Nitrite (CANON) process, which coupled partial nitrification with anammox in a single reactor and thereby achieved efficient single-stage autotrophic nitrogen removal [13,14], and the world’s first anammox engineering plant was successfully commissioned at the Dokhaven wastewater treatment facility in the Netherlands [15], demonstrating the feasibility of applying anammox technology to large-scale wastewater treatment. The implementation of single-stage denitrification processes, as exemplified by the Demonstration of Ammonium Removal Over Nitrite (DEMON), has expanded the application scenarios of anammox technology in the treatment of side-stream wastewater. This stage was focused on studying the physiological properties of the microorganisms and process parameters in order to solve key problems, such as microbial enrichment cultivation and reactor start-up.

The period from 2012 to 2017 represented a mature phase of process optimization and global dissemination for side-stream applications. During this interval, over 100 full-scale anammox plants were commissioned worldwide, demonstrating significant efficacy in treating high-strength nitrogenous streams such as anaerobic digester supernatant and landfill leachate [16]. A defining trend of this era was the technological transition toward mainstream wastewater treatment, marked by the advancement of both two-stage (e.g., SHARON-anammox) and single-stage (e.g., CANON) configurations. While the SHARON process ensured stable nitrite accumulation through stringent operational control, the CANON process offered a more compact footprint by consolidating partial nitrification and anammox within a unified reactor [17]. In China, the landmark 2014 commissioning of large-scale facilities, such as the Beijing Gaobeidian and Kunming Third Wastewater Treatment Plants, signaled the industrial-scale debut of localized anammox solutions [18]. These milestones catalyzed research tailored to China’s specific wastewater characteristics (e.g., low carbon-to-nitrogen ratios), shifting the research frontier toward operational resilience, reactor configuration, and complex interspecies microbial interactions.

The period from 2018 to 2021 represents a pivotal era for advancements in mainstream anammox processes. During this interval, mainstream applications for municipal wastewater treatment gained significant momentum. However, achieving robust anammox bacteria (AnAOB) enrichment and stable operation remains challenging, due to low influent ammonium concentrations and fluctuating low temperatures [19]. To address the bottleneck of unstable nitrite supply, the partial denitrification coupled with anammox (PD/A) process has emerged as a focal point of research. PD/A offers distinct advantages, including reduced organic carbon demand [20], enhanced nitrogen removal efficiency [21], and mitigated sensitivity to substrate limitations and low-temperature inhibition [19]. To further elucidate the underlying microscopic mechanisms, studies have leveraged metagenomics and metabolomics to analyze microbial community structures and functional gene distributions, aiming to decipher the synergistic and competitive interactions between functional taxa [22]. Currently, the research focus has shifted toward the AnAOB and the enhancement of nitrogen removal performance under low-strength substrate conditions. By integrating process innovation with metagenomics and metabolomics, this field is committed to paving a critical pathway for the sustainable and low-energy operation of mainstream wastewater treatment technologies.

The period from 2022 to 2025 is characterized by system integration and intelligent regulation. Driven by the global impetus for a potential carbon-saving benefit transition, nitrogen removal technologies have shifted from energy-intensive, carbon-heavy methods toward paradigms with potential carbon-saving benefits [23]. Anammox has thus evolved beyond mere high-efficiency nitrogen removal to align with the dual goals of energy neutrality and resource recovery [24]. To mitigate greenhouse gas emissions, the integration of iron-mediated nitrate-dependent anaerobic methane oxidation with anammox enables simultaneous nitrogen removal and methane oxidation [25]. Furthermore, coupling anammox with phosphorus recovery (e.g., struvite crystallization) facilitates a holistic transition from pollutant removal to nutrient upcycling [26]. With the advent of Artificial Intelligence, anammox operations are evolving from experience-driven paradigms to data-driven paradigms. This transition, underpinned by multimodal sensors, digital twins, and machine learning algorithms, enables real-time monitoring and precise control over AnAOB metabolic activity and microbial community succession [27]. This current phase prioritizes the physiological responses of functional consortia within real-world wastewater matrices, aiming for a sustainable and intelligent process transformation.

### 4.2. Mechanistic Interpretation and Engineering Implications of Anammox Topic Evolution

Topic 1 affects the practical application and dissemination of this technology in wastewater treatment. Anammox, as a low-energy biological nitrogen removal technology, has shown potential for treating high-ammonia wastewater [28]. The engineering deployment of the anammox process has many limitations, such as the slow growth rate of anammox bacteria, high environmental sensitivity, and long reactor start-up times. To solve these issues, efficient bioreactor configurations and evidence-based start-up procedures allow rapid start-up and stable operation of the process [29]. The declining trend of Topic 1 indicates reduced research attention to fundamental reactor configuration and start-up optimization, but does not by itself demonstrate readiness for routine deployment. Evidence from full-scale operating experience should instead be used to assess whether specific start-up protocols are sufficiently mature for standardization. Accordingly, future work should combine standardized start-up procedures with rapid diagnostic tools and independent operational validation.

Topic 2 focuses on microbial metabolism and regulation. Stable system performance depends on coordinated regulation of anammox bacteria [30]. Metabolic flux can be enhanced by iron ions, signaling molecules, or other agents serving as exogenous electron shuttles or enzymatic cofactors [31]. Anammox bacteria compete with ammonia-oxidizing, nitrite-oxidizing, and heterotrophic denitrifying bacteria in complex niche interactions [32]. Quorum-sensing signaling molecules regulate extracellular polymeric substance synthesis, promoting biofilm formation and interspecies exchange [33]. Multi-omics approaches are increasingly connecting signaling molecules and metabolic genes to community succession, revealing how targeted interventions can control microbial structure and function. The sustained rise in Topic 2 underscores the necessity for wastewater utilities to integrate these insights into systematic microbial monitoring frameworks. By translating omics data into operational indicators, such as functional gene abundance thresholds, utilities can shift from reactive to predictive process management, significantly reducing the risk of performance failure under fluctuating conditions.

Topic 3 addresses mainstream integration. Transitioning anammox from side-stream to mainstream treatment is key, with potential carbon-saving benefits. Compared with side-stream processes, mainstream anammox faces several challenges: low substrate concentration and low temperature [34]. During mainstream anammox treatment of wastewater, dynamic optimization of aeration strategies is employed to balance the metabolic activities of ammonia-oxidizing bacteria and anammox bacteria [35]. Understanding N_2_O diffusion pathways is crucial for optimizing mainstream anammox beyond nitrogen removal [36]. Integrated mainstream anammox systems represent a paradigm shift from simple pollutant removal to climate-friendly operation, balancing nitrogen removal efficiency with carbon footprint optimization. The ascending trajectory of Topic 3 underscores that for water utilities planning carbon-saving upgrades, mainstream integration should be prioritized as the strategic pathway. Specifically, pilot-scale demonstrations, particularly those coupling partial nitritation with intelligent aeration control, offer the most impactful near-term investment for achieving substantial decarbonization in municipal wastewater treatment.

Topic 4 focuses on coupled nitrogen removal technologies. Anammox faces inherent bottlenecks in complex wastewaters: slow growth, environmental stress, and nitrite supply limitations. A multi-pathway coupled denitrification process has been developed to overcome these problems. In situ substrate supply, microbial functional redundancy, and simultaneous multi-pollutant removal have emerged as strategies to advance engineering-scale deployment [37]. The endogenous partial denitrification-phosphorus removal coupled anammox process (SEPD-PR/A) can use endogenous carbon sources to remove carbon, nitrogen, and phosphorus pollutants simultaneously, providing a new way of resource-conserving wastewater treatment [38]. The persistent intensity of Topic 4 indicates sustained research attention to multi-pathway coupling rather than direct evidence of engineering readiness; reported improvements in stability under fluctuating conditions therefore require support from independent experimental and field-scale studies. Future research should prioritize quantifying metabolic flux allocation to refine these intelligent integration strategies, aiming for simultaneous carbon, nitrogen, and phosphorus removal without external carbon addition. For wastewater utilities, especially those managing variable-strength influent, adopting such coupled technologies as a robust design philosophy is essential to achieving efficient nutrient recovery while minimizing greenhouse gas emissions.

Topic 5 concerns process parameter optimization and intelligent control. Online sensor monitoring of NH_4_^+^-N, NO_3_^−^-N, and pH, combined with feedback control based on the NH_4_^+^-N/NO_3_^−^-N ratio, enables real-time adjustment of aeration and influent flow [39]. The literature increasingly discusses a shift from conventional empirical control toward sensor-driven, intelligent decision-making systems. The declining trend of Topic 5 reflects changing research attention rather than demonstrated maturation from manual parameter tuning to field-ready automated control. Integrated sensor networks and data-driven platforms therefore require independent validation of reliability, cost, scalability, energy use, and emissions performance before large-scale application.

### 4.3. Microbial Constraints, Reactor Design, and Operational Strategies for Anammox

Mainstream anammox is an important pathway with potential carbon-saving benefits for municipal wastewater treatment, but its application remains constrained by the characteristics of mainstream wastewater. Low ammonium concentrations and temperatures can limit the growth, activity, and retention of anammox bacteria [40,41]. Biomass-retaining reactor configurations, including granular sludge, biofilm, carrier-based, and membrane-supported systems, together with long-term acclimation, may therefore be advantageous under mainstream conditions. Their performance, however, remains dependent on site-specific influent characteristics and operating conditions [42,43].

NOB represents another major challenge because NOB compete with AnAOB for nitrite and oxidize it to nitrate, reducing substrate availability for anammox [44]. Controlled or intermittent aeration, selective biomass retention, and spatial or functional separation of microbial populations may facilitate NOB suppression, but their effects on ammonia oxidation, anammox activity, and N_2_O emissions must be evaluated concurrently [43,45]. Stable nitrite supply also requires coordinated regulation of ammonia-oxidizing bacteria, AnAOB, and, where applicable, partial-denitrifying populations. To accommodate variations in influent composition, flow, and ammonium loading, influent equalization should be integrated with online monitoring and adaptive control of aeration and flow to maintain microbial and substrate balance [34,46]. These strategies require validation through monitored pilot- and full-scale demonstrations that assess start-up, recovery from disturbances, effluent stability, N_2_O emissions, and life-cycle carbon performance [47].

Although anammox can reduce aeration demand, external carbon input, and sludge production, these advantages do not necessarily translate into net climate benefits. N_2_O has a high global warming potential and can be produced through hydroxylamine-related reactions, nitrifier denitrification mediated by ammonia-oxidizing bacteria, incomplete heterotrophic denitrification, and some abiotic reactions [48,49]. Its emissions are strongly influenced by dissolved oxygen, nitrite accumulation, carbon availability, pH, temperature, loading fluctuations, intermittent aeration, and start-up or recovery phases [50,51]. Because N_2_O exists in both dissolved and gaseous phases, snapshot sampling or off-gas monitoring alone may underestimate total emissions [52,53,54]. Greenhouse gas accounting should also include direct N_2_O emissions from wastewater and sludge treatment, as well as indirect emissions associated with electricity and chemical consumption, sludge transport, treatment, and final disposal [55,56]. Therefore, a unified MRV framework should combine high-frequency liquid-phase and gas-phase monitoring, gas–liquid flow measurements, nitrogen mass balance, and life-cycle accounting to assess the true climate benefits using comparable and verifiable plant-scale data. Successful mainstream anammox demonstrations have primarily been achieved under controlled laboratory, pilot-scale, or site-specific operating conditions. Because low ammonium concentrations, low temperatures, influent variability, NOB competition, and biomass-retention constraints can compromise process stability, long-term full-scale mainstream anammox application remains uncommon in conventional municipal wastewater treatment plants [47,57].

Building on these engineering and environmental challenges, future research should prioritize long-term, cross-seasonal, and multi-site validation of mainstream anammox systems, focusing on start-up, biomass retention, NOB control, nitrite supply, disturbance recovery, and effluent stability. Multi-omics findings should be translated into microbial monitoring indicators, while sensor networks, adaptive control, and digital models should be validated across different reactor configurations and wastewater treatment plants. In addition, plant-wide greenhouse gas MRV, techno-economic analysis, and life-cycle assessment should be conducted, with standardized reporting and cross-plant data sharing used to improve the comparability of results.

### 4.4. Qualitative Appraisal of Deployment-Readiness Evidence

The topic evolution results, considered together with reported engineering evidence, suggest that anammox deployment should be discussed as a staged readiness problem rather than as a uniform technology adoption decision. Side-stream anammox is supported by mature engineering evidence and is most suitable for standardization, operator training, and comparable performance reporting [58,59,60]. Mainstream municipal wastewater treatment remains more uncertain because low ammonium strength, low temperature, variable organic carbon, nitrite-supply instability, and N_2_O emissions can reduce both nitrogen removal reliability and carbon accounting credibility [61,62,63,64]. Coupled anammox-based systems and intelligent control strategies show increasing research momentum, but their implementation requires clearer system boundaries, auditable monitoring data, and site-specific validation before they can support broad wastewater planning with potential carbon-saving benefits (Table 2).

### 4.5. Management Implications for Staged Anammox Deployment

The quantitative evidence presented above suggests that anammox should be treated as a strategic option with potential carbon-saving benefits in wastewater treatment, but not as a universally ready replacement for conventional nitrogen removal [60]. Its management value depends on matching technology maturity with infrastructure conditions, operational capacity, and measurable carbon outcomes [60,75]. The following implications translate the topic-evolution results into priorities for wastewater utilities, regulators, technology providers, and research funders.

From side-stream standardization to mainstream demonstration. Reported full-scale operating experience, rather than the decline in topic intensity alone, supports a relatively mature status for selected side-stream anammox applications. Management actions for this stage should focus on standardizing start-up protocols, operator training, performance reporting, and operational troubleshooting rather than subsidizing repetitive proof-of-concept studies [59]. By contrast, mainstream anammox remains a demonstration-stage application [60]. Municipal systems face low ammonium strength, low temperature, variable organic carbon, nitrite-supply instability, and N_2_O risks [61]. Therefore, implementation should prioritize monitored demonstration projects that compare anammox-based configurations with incumbent nitrification-denitrification under identical performance and carbon-accounting boundaries.

Carbon accounting and N_2_O monitoring as deployment prerequisites. The potential carbon-saving benefits claim of anammox depends on more than reduced aeration and external carbon demand [65]. Without comparable N_2_O accounting, utilities may overestimate climate benefits or transfer emissions from energy consumption to process-related greenhouse gases [76]. Deployment assessment should therefore include monitoring, reporting and verification protocols that cover total nitrogen removal, effluent stability, energy consumption, chemical input, sludge production, direct N_2_O emissions, and life-cycle carbon performance [64,75]. Such protocols are necessary before anammox can be credibly used in potential carbon-saving benefits procurement, wastewater-sector decarbonization plans, or carbon-performance benchmarking [75].

Aligning research and investment with deployment barriers. The growth of topics related to microbial metabolism and mainstream integration indicates increasing research attention to these areas; reported engineering evidence suggests that a key remaining challenge is converting mechanistic understanding into controllable and auditable process performance [63]. Research funding and infrastructure investment should therefore prioritize projects that couple microbial ecology, process control, sensor systems, digital twins, and life-cycle assessment [63,69,75]. Evaluation criteria should include scalability, operational robustness, greenhouse-gas mitigation, and compatibility with existing municipal infrastructure, rather than only laboratory nitrogen-removal rates.

Local validation and technology transfer management. The published evidence base shows that many deployment lessons originate from a limited number of research-intensive settings [47,59]. This creates both an opportunity for knowledge diffusion and a risk of context-insensitive technology transfer. Implementation should be based on open operating data, locally validated design parameters, and capacity building for utilities in regions with weaker research or operational infrastructure [47]. Decision makers should avoid assuming that successful side-stream systems can be directly transplanted into mainstream municipal plants elsewhere without adapting to temperature, influent composition, energy prices, maintenance capacity, financing conditions, and regulatory enforcement [60,61].

A staged implementation roadmap. A staged implementation roadmap is recommended. In the near term, management efforts should support standardization of side-stream operation, disclosure of performance data [59], and baseline carbon accounting [65]. In the medium term, utilities, regulators, and funding agencies should establish mainstream demonstration programs with mandatory N_2_O monitoring and independent life-cycle assessment [75,76]. In the long term, anammox-based systems should be integrated into potential carbon-saving wastewater planning only when their nitrogen-removal reliability and net greenhouse gas benefits are demonstrated under transparent and comparable evaluation frameworks. This staged approach avoids both underinvestment in a promising potential carbon-saving process and premature deployment before climate benefits are verified.

## 5. Study Limitations and Boundary of the Qualitative Readiness Appraisal

Several limitations should be considered when interpreting the qualitative readiness appraisal. First, the dataset was derived from SCIE records in the Web of Science Core Collection. This choice improves bibliographic consistency but may exclude engineering reports, local utility data, government documents, standards, and other grey literature that are highly relevant to technology deployment [77]. Second, DTM and trend indicators capture the direction and concentration of research attention, but they do not directly measure plant-level readiness, cost-effectiveness, regulatory feasibility, or public acceptability. Third, topic modelling identifies thematic structures from published text and therefore depends on how authors describe their work. This qualitative framework cannot replace field-scale performance evaluation, techno-economic analysis, life-cycle assessment, or formal TRL evaluation [75,78]. Fourth, because the trend regressions were based on a limited number of annual observations, the estimated slopes and statistical significance may be sensitive to individual years, short-term fluctuations, temporal autocorrelation, and non-linear patterns. Accordingly, the regression results were used only to describe the overall direction of changes in research attention and should not be used for causal inference, long-term trend forecasting, or technology-readiness assessment. Recent changes should be regarded as exploratory signals and verified using longer time series and other independent evidence.

These limitations define the boundary of the deployment claims advanced here. The study does not argue that rising research intensity alone should trigger broad implementation of anammox. Rather, it uses topic evolution evidence together with reported engineering studies to qualitatively identify where management action may be better supported, where demonstration and MRV are required, and where further research should reduce uncertainty before deployment. Local decisions will still need to account for influent characteristics, climate, energy mix, operator capacity, regulatory enforcement, and financing conditions [47,61].

## 6. Conclusions

This study used dynamic topic modelling and an evidence-based qualitative appraisal to examine the evolution of anammox research and organize deployment-related evidence. Research priorities have gradually shifted from reactor start-up and process optimization towards microbial regulation, mainstream integration, coupled nitrogen-removal processes, and intelligent control. These shifts reflect changes in research attention rather than evidence of formal technological maturity. Anammox can reduce aeration and external organic-carbon demands, thereby offering potential energy and carbon-saving benefits. Side-stream applications are supported by stronger full-scale engineering evidence and are therefore better positioned for standardization and comparable performance reporting, whereas mainstream applications remain constrained by low ammonium concentrations, low temperatures, NOB competition, biomass retention, and unstable nitrite supply. Addressing these limitations requires monitored multi-site demonstrations, standardized accounting of N_2_O and plant-wide greenhouse gas accounting, life-cycle and techno-economic assessments, and transparent plant-scale operating data. The climate benefits of anammox are conditional rather than inherent, and wider implementation should proceed through staged, site-specific, and evidence-based decisions supported by a credible MRV framework.

## Figures and Tables

**Table 1:** Topic word distribution.

Topic Number	Topic Name	Key Vocabulary (Top 10)
Topic 1	Reactor configuration and start-up	reactor, sludge, bacteria, temperature, biofilm, rate, low, concentration, treatment, start
Topic 2	Microbial metabolism and regulation	activity, performance, sludge, microbial, bacteria, concentration, system, addition, efficiency, reactor
Topic 3	Mainstream process integration	bacteria, system, treatment, microbial, membrane, oxidation, energy, efficient, model, partial
Topic 4	Coupled nitrogen removal technologies	denitrification, partial, organic, carbon, bacteria, cod, sludge, simultaneous, system, treatment
Topic 5	Process parameters and control	reactor, partial, concentration, treatment, rate, cod, system, influent, nitrification, efficiency

**Table 2:** Qualitative deployment-readiness evidence matrix for anammox with potential carbon-saving benefits in wastewater treatment.

Application Stage	Main Deployment Risks	Carbon-Accounting Requirements	Management Actions	References
Side-stream anammox	Start-up reliability, operator expertise, reporting comparability	Energy use, chemical inputs, sludge production, baseline N_2_O checks	Standardize start-up and operation; disclose comparable performance data; strengthen operator training	[58,59,65]
Mainstream municipal wastewater	Low ammonium strength, low temperature, variable loads, nitrite-supply instability	Direct N_2_O monitoring, whole-process LCA, comparison with incumbent nitrification-denitrification	Develop monitored pilot programs; use independent evaluation; avoid broad deployment before site-specific validation	[47,62,63,64]
Coupled anammox-based systems	Process coordination, multi-pollutant trade-offs, control complexity	System-boundary definition, energy-carbon trade-offs, nutrient-recovery accounting	Assess integration with existing infrastructure; compare nitrogen, carbon, and co-pollutant performance	[66,67,68]
Anammox with intelligent control	Sensor reliability, data availability, model transferability, maintenance capacity	MRV-compatible operating data, digital records, real-time N_2_O and energy indicators	Link digital control with audit-ready monitoring; require transparent data governance	[69,70,71,72]
Wastewater planning with carbon-saving objectives	Premature carbon claims, financing and regulatory constraints	Plant-level LCA, net greenhouse-gas benefit, uncertainty reporting	Use staged deployment, standards, and funding tied to verified performance	[54,65,73,74,75]

## Data Availability

The original contributions presented in this study are included in the article/Supplementary Materials. Further inquiries can be directed to the corresponding author.

## Supplementary Materials

The following supporting information can be downloaded at: https://www.mdpi.com/article/10.3390/microorganisms14092020/s1, Figure S1: Schematic architecture of the dynamic topic modeling (DTM); Table S1: Ranking of the top 20 keywords with the highest weights (2001–2011); Table S2: Ranking of the top 20 keywords with the highest weights (2012–2017); Table S3: Ranking of the top 20 keywords with the highest weights (2018–2021); Table S4: Ranking of the top 20 keywords with the highest weights (2022–2025).

## Abbreviations

The following abbreviations are used in this manuscript:

Anammox	Anaerobic ammonium oxidation
AnAOB	Anaerobic ammonium-oxidizing bacteria
CANON	Completely Autotrophic Nitrogen removal Over Nitrite
DTM	Dynamic topic modelling
LCA	Life-cycle assessment
MRV	Monitoring, reporting, and verification
N_2_O	Nitrous oxide
PD/A	Partial denitrification/anammox
SCIE	Science Citation Index Expanded
WoSCC	Web of Science Core Collection
