# Supplementary material for: Deployment Readiness of Anammox for Wastewater Treatment with Potential Carbon-Saving Benefits: Environmental Risks, Monitoring Requirements and Implementation Pathways
Source: Microorganisms. 2026 Sep 11;14(9):2020. doi: 10.3390/microorganisms14092020 (PMC13609761; doi:10.3390/microorganisms14092020)
Supplement: Supplementary file 1 [file microorganisms-14-02020-s001.zip › microorganisms-4467236-supplementary.pdf]

Supplementary Materials

Deployment readiness of anammox for wastewater treatment with potential carbon-saving benefits: environmental risks, monitoring requirements and implementation pathways

Figure S1 Schematic architecture of the dynamic topic modeling (DTM)

Table S1 Ranking of the top 20 keywords with the highest weights (2001-2011).

Table S2 Ranking of the top 20 keywords with the highest weights (2012-2017).

Table S3 Ranking of the top 20 keywords with the highest weights (2018-2021).

Table S4 Ranking of the top 20 keywords with the highest weights (2022-2025).

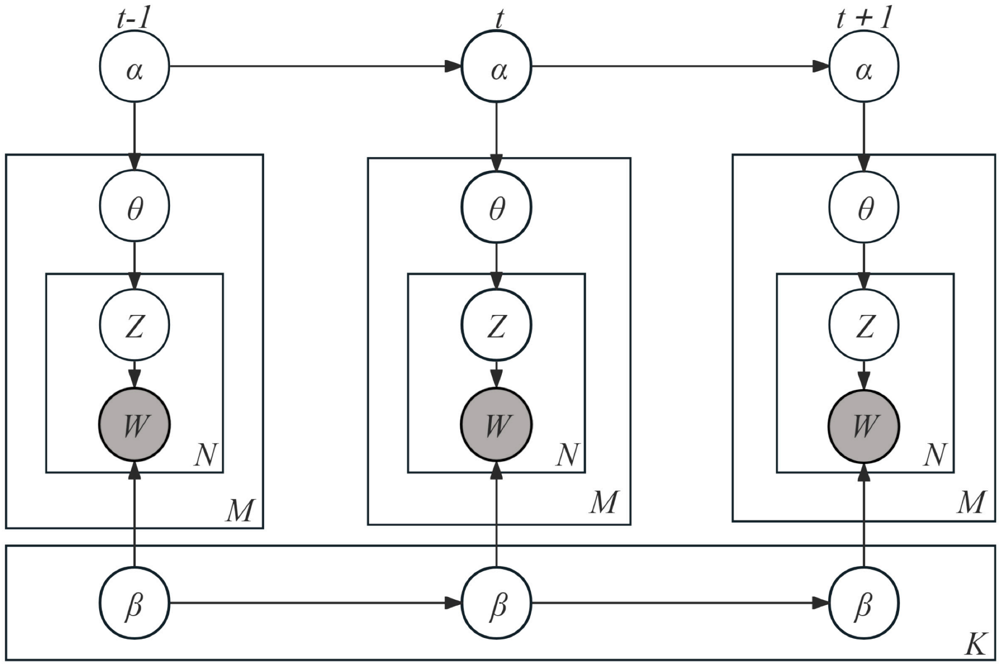

Figure S1 Schematic architecture of the dynamic topic modeling (DTM). *K* denotes the number of latent topics; *M* represents the number of documents within a specific time slice; and *N* signifies the number of words in a given text. *Z* and *W* correspond to the assigned topics and the observed words, respectively. The hyperparameters α and β represent the Dirichlet priors for the document-topic distribution (θ) and the topic-word distribution, respectively. θ characterizes the specific topic proportions assigned to each document across consecutive time slices

Table S1 Ranking of the top 20 keywords with the highest weights (2001-2011)

Rank	Topic1		Topic2		Topic3		Topic4		Topic5	
	word	weight	word	weight	word	weight	word	weight	word	weight
1	reactor	0.0357	activity	0.0187	bacteria	0.0281	denitrification	0.0435	reactor	0.0348
2	sludge	0.0280	performance	0.0177	treatment	0.0159	organic	0.0225	partial	0.0241
3	bacteria	0.0265	sludge	0.0155	system	0.0156	partial	0.0223	concentration	0.0206
4	temperature	0.0259	microbial	0.0135	microbial	0.0141	carbon	0.0219	treatment	0.0196
5	biofilm	0.0200	bacteria	0.0133	membrane	0.0131	bacteria	0.0200	rate	0.0169
6	rate	0.0194	concentration	0.0128	oxidation	0.0122	cod	0.0155	cod	0.0142
7	low	0.0173	addition	0.0114	model	0.0103	sludge	0.0146	influent	0.0133
8	concentration	0.0136	system	0.0111	energy	0.0100	simultaneous	0.0126	system	0.0131
9	treatment	0.0127	reactor	0.0109	efficient	0.0098	treatment	0.0125	nitrification	0.0127
10	start	0.0118	efficiency	0.0109	methane	0.0094	efficiency	0.0125	efficiency	0.0126
11	activity	0.0112	oxidation	0.0101	damo	0.0089	system	0.0123	oxidation	0.0113
12	biomass	0.0089	salinity	0.0100	partial	0.0089	anoxic	0.0118	sludge	0.0110
13	performance	0.0089	inhibition	0.0093	mainstream	0.0088	low	0.0107	oxygen	0.0107
14	efficiency	0.0083	treatment	0.0086	low	0.0083	phosphorus	0.0098	leachate	0.0100
15	mainstream	0.0080	extracellular	0.0085	efficiency	0.0081	nitrification	0.0097	bacteria	0.0093
16	operation	0.0080	gene	0.0081	rate	0.0077	denitrify	0.0097	low	0.0091
17	carrier	0.0078	substance	0.0081	biofilm	0.0077	source	0.0085	oxidize	0.0087
18	candidatus	0.0075	recovery	0.0079	reactor	0.0071	novel	0.0079	autotrophic	0.0078
19	community	0.0072	stress	0.0078	community	0.0066	candidatus	0.0075	landfill	0.0077
20	oxidation	0.0071	functional	0.0078	oxidize	0.0062	biofilm	0.0070	denitrification	0.0071

Table S2 Ranking of the top 20 keywords with the highest weights (2012-2017)

Rank	Topic1		Topic2		Topic3		Topic4		Topic5	
	word	weight	word	weight	word	weight	word	weight	word	weight
1	reactor	0.0356	activity	0.0188	bacteria	0.0284	denitrification	0.0441	reactor	0.0348
2	sludge	0.0281	performance	0.0178	system	0.0159	partial	0.0227	partial	0.0247
3	bacteria	0.0266	sludge	0.0157	treatment	0.0158	organic	0.0226	concentration	0.0209
4	temperature	0.0262	microbial	0.0138	microbial	0.0143	carbon	0.0221	treatment	0.0196
5	biofilm	0.0202	bacteria	0.0135	membrane	0.0132	bacteria	0.0203	rate	0.0168
6	rate	0.0194	concentration	0.0129	oxidation	0.0122	cod	0.0156	cod	0.0143
7	low	0.0186	addition	0.0114	model	0.0103	sludge	0.0146	influent	0.0134
8	concentration	0.0136	system	0.0112	energy	0.0101	simultaneous	0.0128	system	0.0133
9	treatment	0.0128	efficiency	0.0110	efficient	0.0098	efficiency	0.0126	nitrification	0.0130
10	start	0.0119	reactor	0.0109	methane	0.0094	system	0.0125	efficiency	0.0127
11	activity	0.0112	salinity	0.0102	mainstream	0.0089	treatment	0.0124	oxidation	0.0113
12	performance	0.0089	oxidation	0.0102	partial	0.0089	anoxic	0.0118	sludge	0.0109
13	biomass	0.0088	inhibition	0.0094	damo	0.0089	low	0.0109	oxygen	0.0108
14	efficiency	0.0084	treatment	0.0086	low	0.0084	nitrification	0.0100	leachate	0.0100
15	mainstream	0.0083	extracellular	0.0085	efficiency	0.0082	denitrify	0.0098	bacteria	0.0094
16	operation	0.0080	gene	0.0083	rate	0.0078	phosphorus	0.0097	low	0.0093
17	carrier	0.0078	substance	0.0080	biofilm	0.0078	source	0.0085	oxidize	0.0088
18	candidatus	0.0076	recovery	0.0080	reactor	0.0071	novel	0.0079	landfill	0.0079
19	community	0.0072	functional	0.0079	community	0.0067	candidatus	0.0076	autotrophic	0.0078
20	oxidation	0.0071	investigate	0.0078	oxidize	0.0062	biofilm	0.0070	remove	0.0072

Table S3 Ranking of the top 20 keywords with the highest weights (2018-2021)

Rank	Topic1		Topic2		Topic3		Topic4		Topic5	
	word	weight	word	weight	word	weight	word	weight	word	weight
1	reactor	0.0354	activity	0.0189	bacteria	0.0287	denitrification	0.0448	reactor	0.0348
2	sludge	0.0287	performance	0.0178	treatment	0.0159	partial	0.0232	partial	0.0254
3	bacteria	0.0272	sludge	0.0160	system	0.0157	organic	0.0226	concentration	0.0211
4	temperature	0.0265	microbial	0.0141	microbial	0.0145	carbon	0.0221	treatment	0.0196
5	low	0.0209	bacteria	0.0141	membrane	0.0132	bacteria	0.0207	rate	0.0169
6	biofilm	0.0206	concentration	0.0128	oxidation	0.0123	cod	0.0156	cod	0.0145
7	rate	0.0192	addition	0.0115	energy	0.0102	sludge	0.0148	system	0.0135
8	concentration	0.0136	system	0.0115	model	0.0101	simultaneous	0.0127	nitrification	0.0135
9	treatment	0.0128	efficiency	0.0112	efficient	0.0100	system	0.0127	influent	0.0134
10	start	0.0121	reactor	0.0109	methane	0.0093	efficiency	0.0127	efficiency	0.0129
11	activity	0.0116	salinity	0.0105	mainstream	0.0091	treatment	0.0125	oxidation	0.0113
12	performance	0.0090	oxidation	0.0101	partial	0.0090	anoxic	0.0118	oxygen	0.0110
13	mainstream	0.0087	inhibition	0.0093	damo	0.0087	low	0.0110	sludge	0.0109
14	biomass	0.0086	treatment	0.0087	low	0.0086	nitrification	0.0102	leachate	0.0101
15	efficiency	0.0084	extracellular	0.0086	efficiency	0.0082	denitrify	0.0101	bacteria	0.0096
16	operation	0.0080	gene	0.0084	biofilm	0.0078	phosphorus	0.0095	low	0.0095
17	candidatus	0.0077	substance	0.0081	rate	0.0078	source	0.0085	oxidize	0.0090
18	carrier	0.0077	functional	0.0081	reactor	0.0071	novel	0.0081	landfill	0.0080
19	community	0.0074	recovery	0.0081	community	0.0067	candidatus	0.0077	autotrophic	0.0080
20	oxidation	0.0070	low	0.0079	oxidize	0.0063	biofilm	0.0071	remove	0.0073

Table S4 Ranking of the top 20 keywords with the highest weights (2022-2025)

Rank	Topic1		Topic2		Topic3		Topic4		Topic5	
	word	weight	word	weight	word	weight	word	weight	word	weight
1	reactor	0.0353	activity	0.0190	bacteria	0.0288	denitrification	0.0455	reactor	0.0348
2	sludge	0.0294	performance	0.0177	system	0.0166	partial	0.0236	partial	0.0256
3	temperature	0.0267	sludge	0.0165	treatment	0.0161	organic	0.0227	concentration	0.0211
4	bacteria	0.0265	microbial	0.0144	microbial	0.0146	carbon	0.0222	treatment	0.0197
5	low	0.0230	bacteria	0.0136	membrane	0.0132	bacteria	0.0210	rate	0.0169
6	biofilm	0.0212	concentration	0.0127	oxidation	0.0122	cod	0.0156	cod	0.0147
7	rate	0.0190	system	0.0116	energy	0.0102	sludge	0.0150	system	0.0137
8	concentration	0.0136	addition	0.0116	efficient	0.0101	system	0.0129	influent	0.0135
9	treatment	0.0129	efficiency	0.0115	model	0.0098	efficiency	0.0128	nitrification	0.0132
10	start	0.0123	reactor	0.0108	partial	0.0092	treatment	0.0127	efficiency	0.0130
11	activity	0.0114	salinity	0.0101	methane	0.0092	simultaneous	0.0120	oxidation	0.0113
12	mainstream	0.0090	oxidation	0.0100	mainstream	0.0090	anoxic	0.0117	oxygen	0.0112
13	performance	0.0090	inhibition	0.0092	low	0.0088	low	0.0112	sludge	0.0109
14	biomass	0.0085	extracellular	0.0089	damo	0.0084	denitrify	0.0100	low	0.0098
15	efficiency	0.0085	treatment	0.0088	efficiency	0.0083	nitrification	0.0099	bacteria	0.0097
16	operation	0.0081	gene	0.0086	biofilm	0.0078	phosphorus	0.0090	leachate	0.0094
17	carrier	0.0080	substance	0.0083	rate	0.0077	source	0.0085	oxidize	0.0091
18	candidatus	0.0079	functional	0.0082	reactor	0.0071	novel	0.0082	autotrophic	0.0081
19	community	0.0073	stress	0.0081	community	0.0067	candidatus	0.0079	landfill	0.0077
20	oxidation	0.0069	recovery	0.0081	oxidize	0.0063	biofilm	0.0076	denitrification	0.0074
